# The juxtaposition of *Ilex cornuta* fruit and gut microbiota against alcoholic liver disease based on the integrated pharmacology via metabolomics

**DOI:** 10.1002/ctm2.1392

**Published:** 2023-09-05

**Authors:** Ki‐Kwang Oh, Sang‐Jun Yoon, Su‐Been Lee, Sang Youn Lee, Haripriya Gupta, Raja Ganesan, Satya Priya Sharma, Sung‐Min Won, Jin‐Ju Jeong, Dong Joon Kim, Ki‐Tae Suk

**Affiliations:** ^1^ Institute for Liver and Digestive Diseases, College of Medicine Hallym University Chuncheon South Korea

Dear Editor,

We integrated significant bio‐database platforms with microbial informatics to pioneer the therapeutic agents of alcoholic liver disease (ALD) in a toolbox, suggesting that the toolbox can be utilized as a component to discover potential bioactive(s) against ALD.

Over the past decades, ALD has emerged the leading cause of mortality, announcing that alcohol‐associated liver cirrhosis (LC) gave birth to approximately 600 000 deaths in 2016.^1^ The intemperate alcohol consumption is a main cause of ALD, which can advance to hepatocellular carcinoma (HCC). According to recent study, the aqueous extraction of *Ilex cornuta* leaves (ICLs) was an important prescription to alleviate high‐fat‐diet (HFD) in an animal experiment.^2^ Comparatively, herbal leaves and fruits contained key bioactive compounds, but fruits possess more distinctive chemicals and had more plentiful bioactives than leaves.^3^ Based on the theory, *Ilex cornuta* fruit (ICF) was considered as research source, its pharmacological mechanism is yet to be clarified. Recently, a report demonstrated that gut microbiota (GM) plays central role to ameliorate fatty liver diseases, including ALD.^4^ Based on the scientific evidence, we postulated that the orchestration of ICF and metabolites from GM might make positive effects against ALD. With the exactness and rigor, bioinformatics, cheminformatics, microbial informatics, and computer‐aided drug screening tool were employed to implement the investigation. The workflow is represented in Figure 1.

**FIGURE 1:**
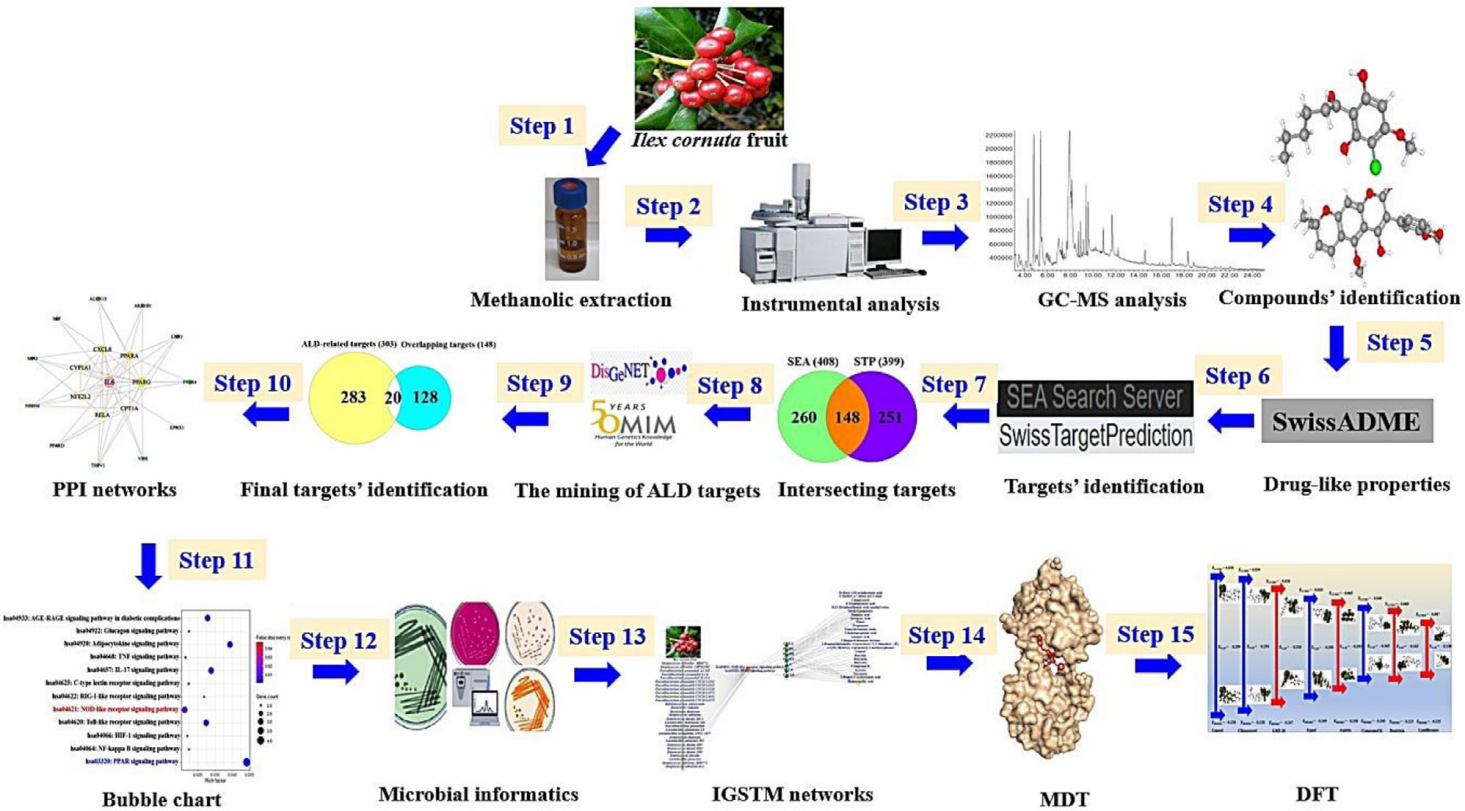
The workflow of this study.

The methanolic extraction of ICF was utilized to analyze gas chromatography mass spectrometry (GC‐MS), identified 30 molecules according to retention time (Table S1). The ICF and its GC‐MS chromatogram were displayed in Figure 2A,B, two key molecules were indicated in the diagram. All molecules were the potential drug candidates in physicochemical properties (Supplementary Table S2). The corresponding targets linked to 30 molecules from ICF were selected by similarity ensemble approach (SEA) (408 targets),^5^ and SwissTargetPrediction (STP) (399 targets)^6^ (Table S3). The number of 148 intersecting targets was selected via the comparative analysis on Venn diagram (Supplementary Table S3), (Figure 2C). Then, ALD‐responded targets (303 targets) (Table S3) were gathered via DisGeNET,^7^ and OMIM.^8^ The Venn diagram exhibited the 20 targets (Supplementary Table S3) by comparing the 148 overlapping targets and ALD‐associated 303 targets (Figure 2D), the selected 20 targets were defined as ‘uppermost targets’. The 20 uppermost targets were input into STRING database to construct protein‐protein interaction (PPI) networks to clarify possible signaling pathways of ICF against ALD. The STRING database exhibited as the 19 nodes (the number of targets) and 56 edges (the number of connectivity) (Figure 2E), notably, GSTK1 had no connectivity with neighbor targets. In the 19 targets, IL6 (16) with the highest degree value (DV) was allowed for a key target, followed by PPARG (13), PPARA (11), and CXCL8 (10). Also, the ranking of betweenness centrality (BC) was the same as the DV order: IL6 (1), PPARG (.38), PPARA (.14), and CXCL8 (.06) (Table S4). Thereby, a bubble chart shows a total of 12 signaling pathways linked directly to ALD (Figure 2F), (Table S5), which was confirmed as agonism, or antagonism based on rich factor (RF) of each signaling pathway. The gutMGene^9^ as microbial informatics was adopted to identify GM related to the two signaling pathways (the highest or lowest signaling pathway). In the 12 signaling pathways, PPAR signaling pathway ranked the greatest RF was defined as an agonism, and nucleotide‐binding oligomerization domain (NOD)‐like receptor signaling pathway ranked the smallest RF was characterized as an antagonism.

FIGURE 2(A) The picture of *Ilex cornuta* fruit (ICF). (B) The gas chromatography mass spectrometry (GC‐MS) of ICF. (C) The overlapping targets between SEA and STP. (D) The final targets against alcoholic liver disease (ALD). (E) PPI networks. (F) The number of 12 signaling pathways related to occurrence and progression of ALD. Red text: Antagonistic mechanism; Blue text: Agonistic mechanism. (G) ICF or gut microbiota (GM)‐signaling pathways‐targets‐metabolites (IGSTM) networks.

**Figure:**
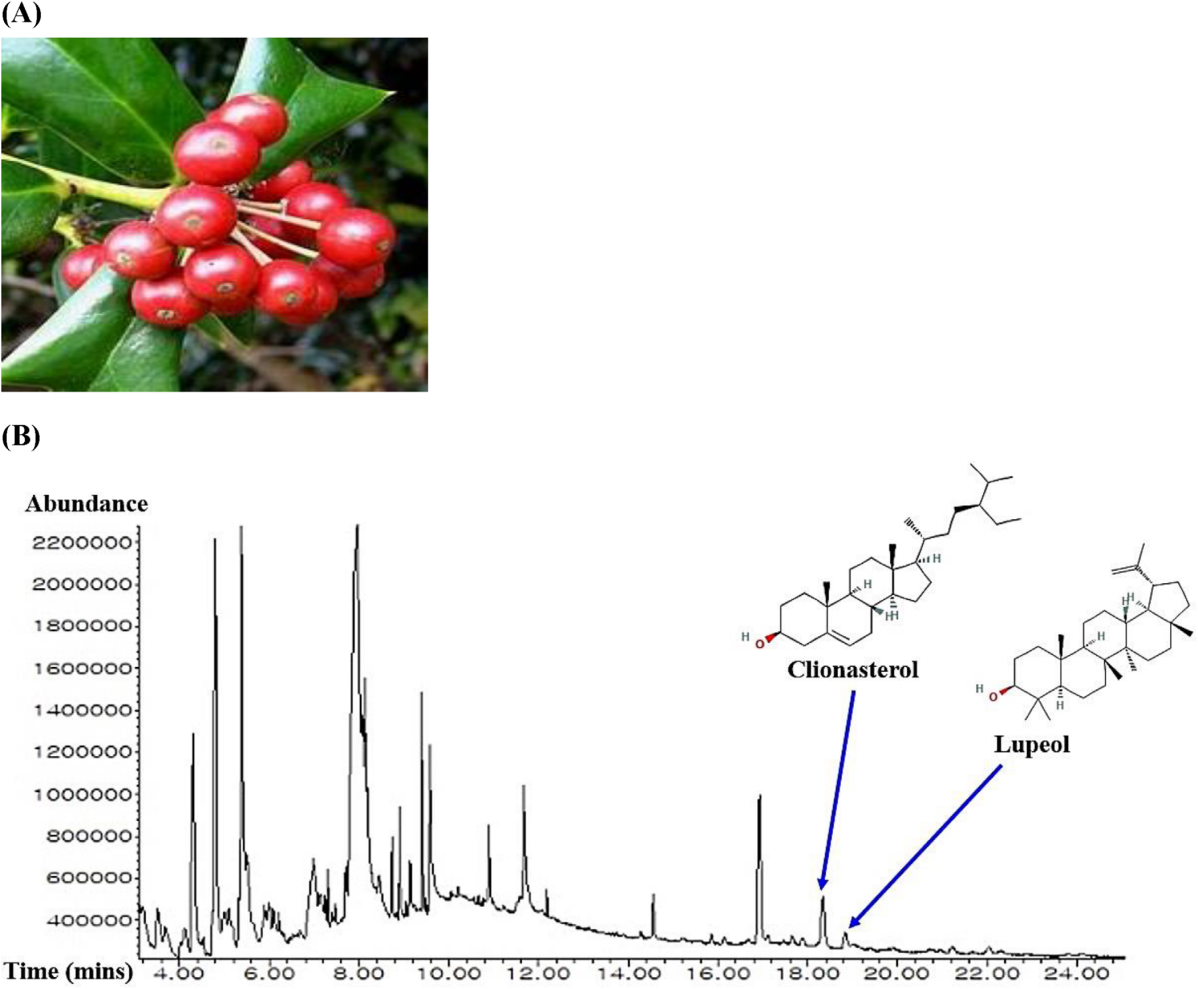

**Figure:**
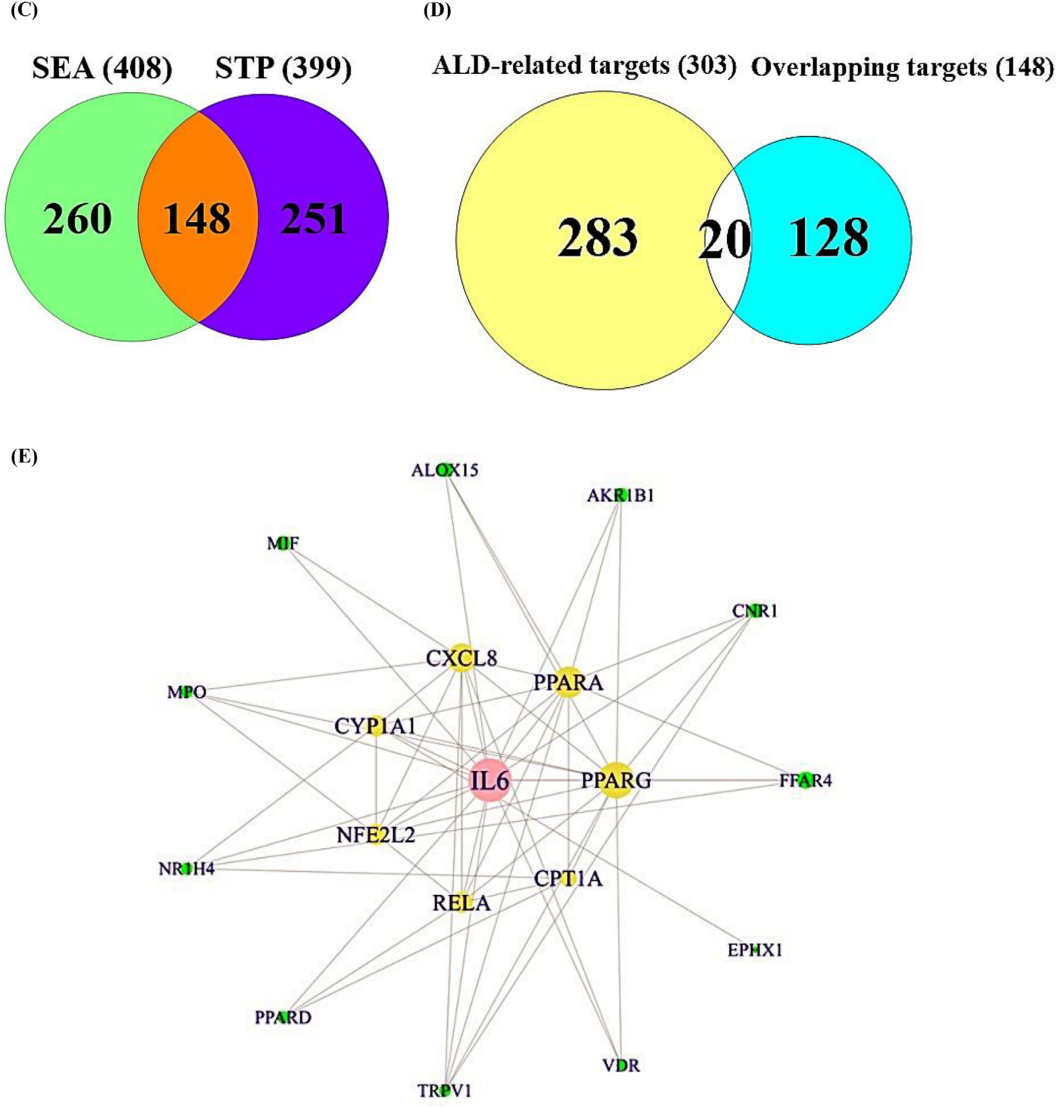

**Figure:**
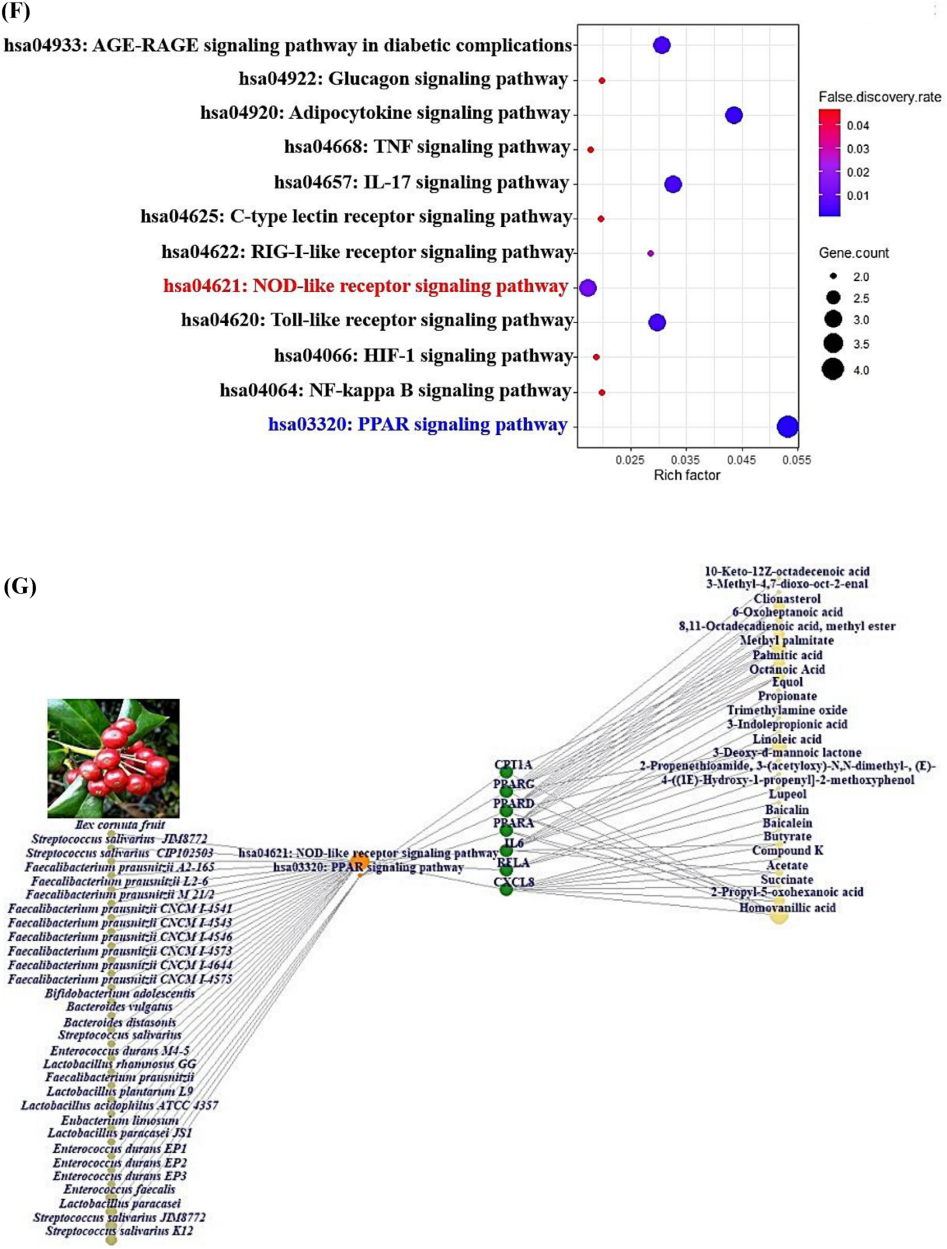

The ICF or GM‐Signaling pathways ‐Targets—Metabolites (IGSTM) networks show that ICF, 29 GM, two signaling pathways, seven targets, and 25 metabolites were involved in alleviating ALD, with 64 nodes, and 85 edges (Figure 2G). In detail, the PPAR signaling pathway was related to four GM, 11 metabolites, and four targets (PPARA, PPARD, PPARG, and CPT1A). Then, the NOD‐like receptor signaling pathway was associated with 25 GM, 17 metabolites, and three targets (CXCL8, RELA, and IL6). In the NOD‐like receptor signaling pathway, the Molecular Docking Test (MDT) uncovered that Compound K‐CXCL8 conformer from GM formed the most stabilized complex with the lowest binding energy (─8.9 kcal/mol), which had better affinity than a standard drug (Danirixin: ─8.3 kcal/mol). However, the GM to produce Compound K is yet to be reported. Then, Lupeol‐RELA conformer (─7.6 kcal/mol) from ICF, and Equol‐IL6 conformer (─7.4 kcal/mol) from *Lactobacillus paracasei JS1* had more optimal affinity than standard drugs (Aspirin, LMT‐28). In the PPAR signaling pathway, MDT revealed that Clionasterol had pan‐PPAR agonistic properties, which might have better affinity than Lanifibranor known as a pan‐PPAR agonist. However, the CPT1A target had no valid conformer with any metabolites. The MDT information is represented in Supplementary Table S6, Figure 3A–F. Checking the donor or acceptor on its chemical bond(s) to the compound can be approved by energy gap via the highest occupied molecular orbital (HOMO) and lowest unoccupied molecular orbital (LUMO).^10^ The four key molecules (Lupeol, Clionasterol, Equol, and Compound K) had significant HOMO values (─.230 kcal/mol, ─.228 kcal/mol, ─.209 kcal/mol, and ─.205 kcal/mol) and might be considerable electron donors in comparison to standard compounds (LMT‐28: ─.247 kcal/mol, Aspirin: ─.258 kcal/mol, Danirixin: ─.223 kcal/mol, and Lanifibranor: ─.225 kcal/mol), respectively. The stability of a molecule was dependent upon energy gap between HOMO and LUMO, and d lower energy gap can be determined as a soft compound with favorable reactivity (Table S7). Of the four key compounds, Compound K showed the most reactivity, which had similar characteristics to the standard molecule. The detailed pattern of density functional theory (DFT) is represented in Figure 3G.

FIGURE 3(A) CXCL8‐Compound K conformer. (B) RELA‐Lupeol conformer. (C) IL6‐Equol conformer. (D) PPARA‐Clionasterol. (E) PPARD‐Clionasterol. (F) PPARG‐Clionasterol. (G) The energy gap (HOMO − LUMO) plots of key molecules of *Ilex cornuta* fruit (ICF), metabolites of gut microbiota (GM), and standard drugs.

**Figure:**
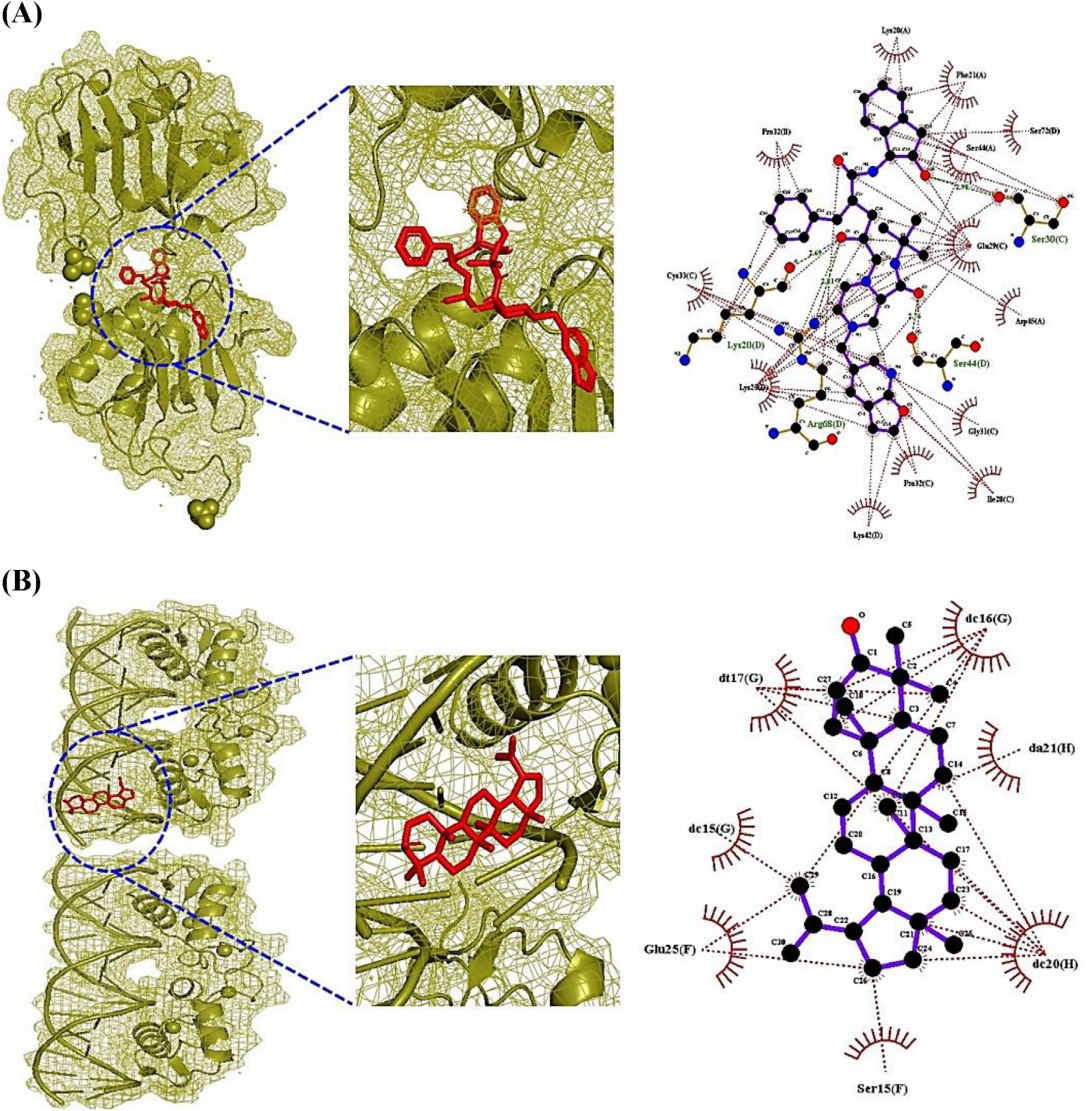

**Figure:**
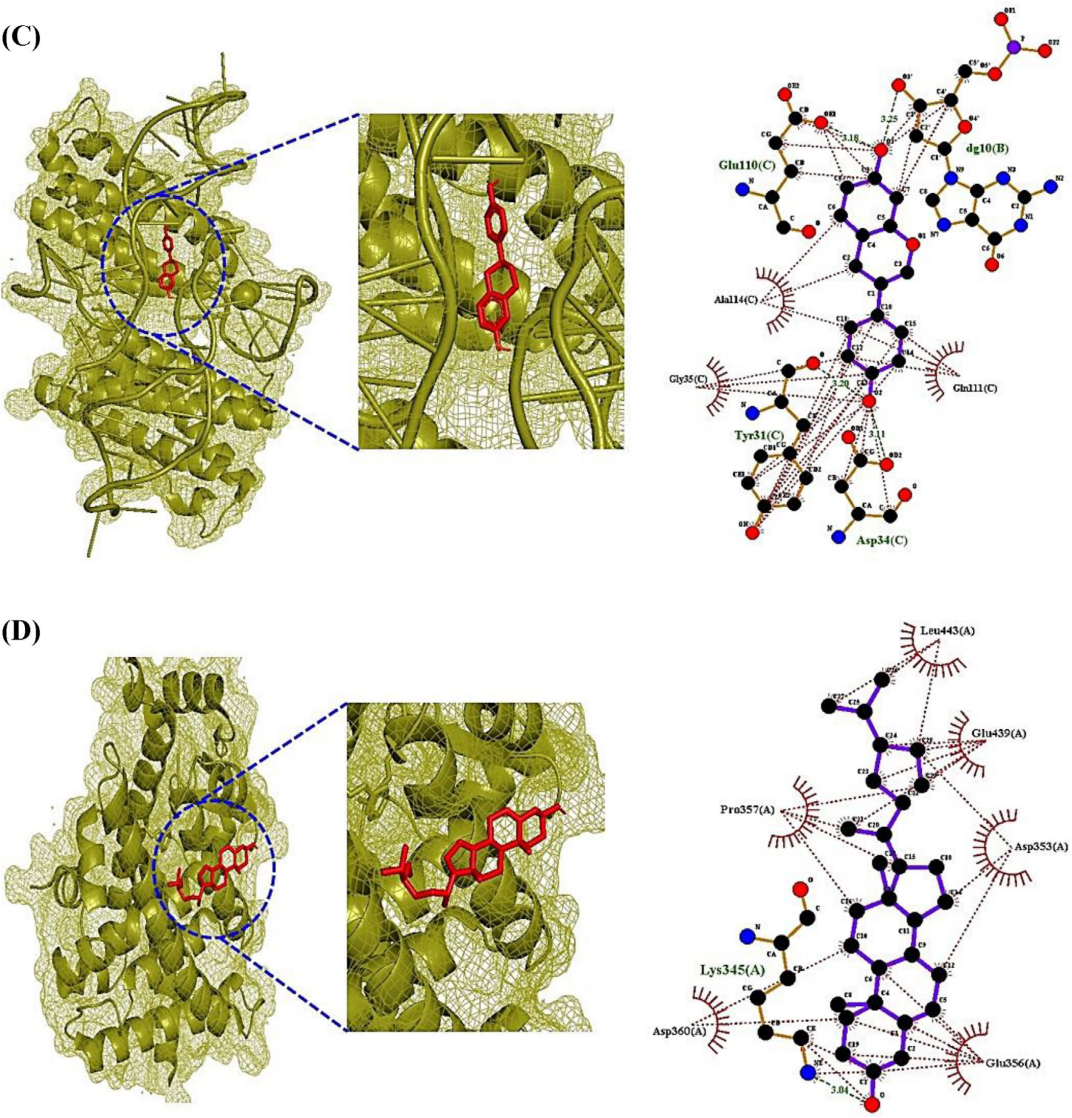

**Figure:**
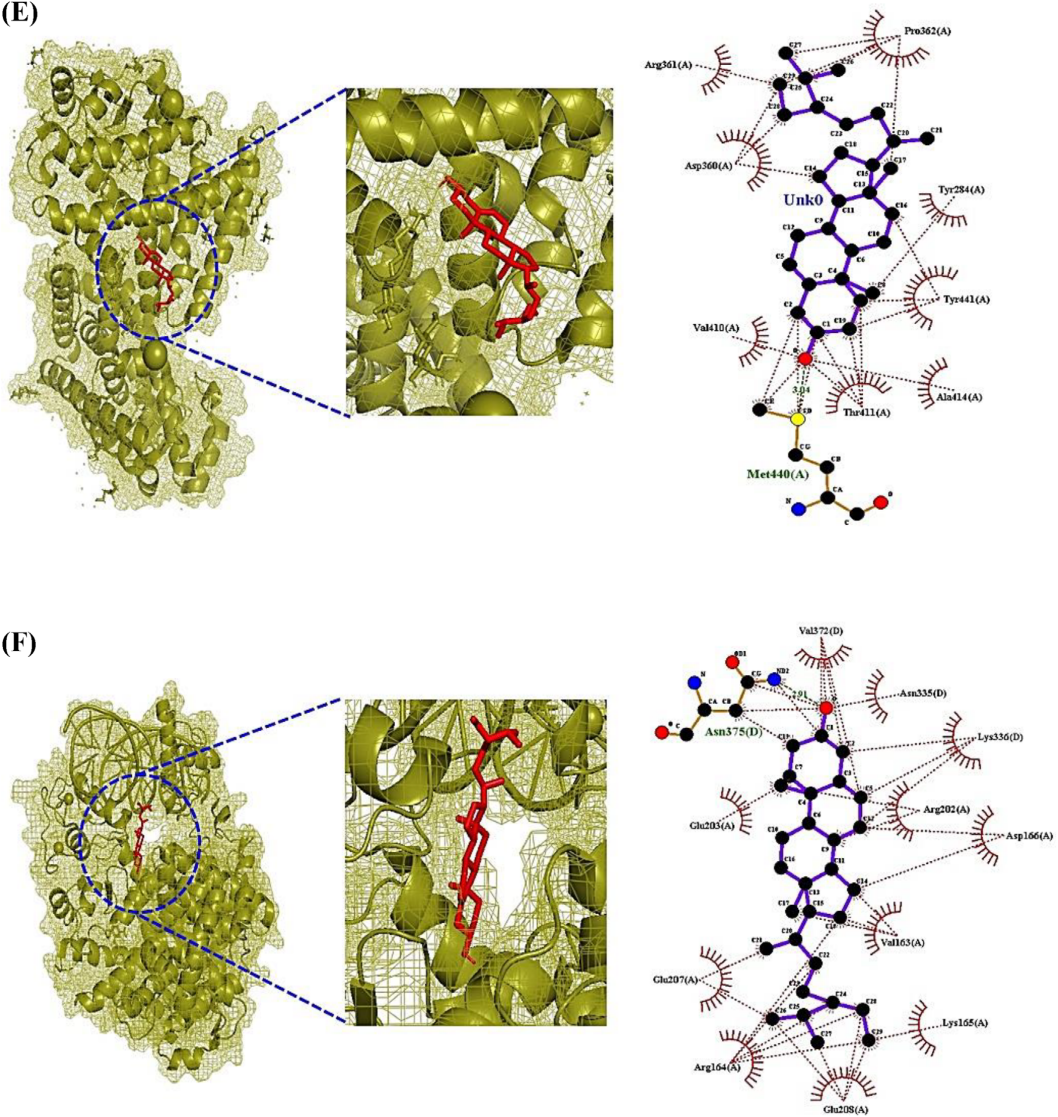

**Figure:**
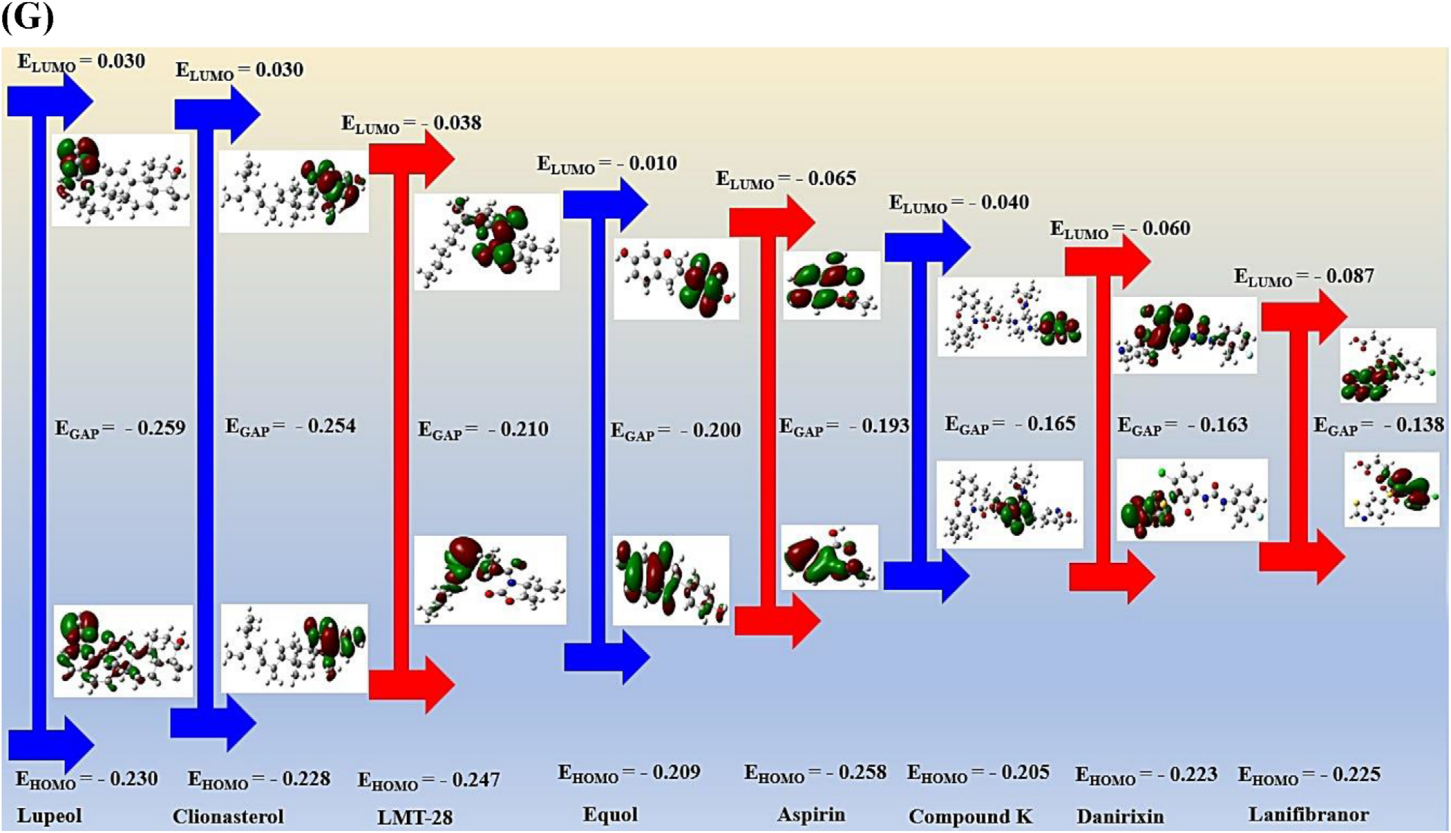

The findings suggest that all experimental molecules (Lupeol, Clionasterol, Equol, and Compound K) had better kinetic binding stability than positive controls (LMT‐28, Aspirin, Danirixin, and Lanifibranor). This scenario illustrated that the complicated microbiome atlas between endogenous species (or GM) and exogenous species (or ICF or diet) can be decrypted by conducting biodata fusion. This study represented that antagonistic conformers of NOD‐like receptor signaling pathway are CXCL8‐Compound K, RELA‐Lupeol, and IL6‐Equol; agonistic conformers of PPAR signaling pathway are PPARA, PPARD, PPARG‐Clionasterol via the toolbox. Overall, this study supports scientific evidence that ICF and GM can orchestrate to exert favorable effects against ALD.

## CONFLICT OF INTEREST STATEMENT

The authors declare that they have no competing interests.

## FUNDING INFORMATION

Hallym University Research Fund, The Basic Science Research Program through the National Research Foundation of Korea (NRF), The Ministry of Education, Science and Technology, Grant Numbers: NRF2019R1I1A3A01060447 and NRF‐2020R1A6A1A03043026; Korea Institute for Advancement of Technology, Grant Number: P0020622; Bio Industrial Technology Development Program, The Ministry of Trade, Industry and Energy (MOTIE, Korea), Grant Number: 20018494

## Supporting information

Supporting Information

Supporting Information

Supporting Information

Supporting Information

Supporting Information

Supporting Information

Supporting Information

## Data Availability

The datasets utilized and/or analyzed during the current study are available from the corresponding author upon reasonable request.
